# Therapeutic Blockade of Immune Complex-Mediated Glomerulonephritis by Highly Selective Inhibition of Bruton’s Tyrosine Kinase

**DOI:** 10.1038/srep26164

**Published:** 2016-05-19

**Authors:** Samantha A. Chalmers, Jessica Doerner, Todd Bosanac, Sara Khalil, Dustin Smith, Christian Harcken, Janice Dimock, Evan Der, Leal Herlitz, Deborah Webb, Elise Seccareccia, Di Feng, Jay S. Fine, Meera Ramanujam, Elliott Klein, Chaim Putterman

**Affiliations:** 1 Albert Einstein College of Medicine, Bronx, NY, USA; 2 Small Molecule Discovery Research, Boehringer Ingelheim Pharmaceuticals, Ridgefield, CT, USA; 3 Immunology and Respiratory Disease Research, Boehringer Ingelheim Pharmaceuticals, Ridgefield, CT, USA; 4 Cleveland Clinic, Cleveland, OH, USA; 5 Computational Biology, Boehringer Ingelheim Pharmaceuticals, Ridgefield, CT, USA

## Abstract

Lupus nephritis (LN) is a potentially dangerous end organ pathology that affects upwards of 60% of lupus patients. Bruton’s tyrosine kinase (BTK) is important for B cell development, Fc receptor signaling, and macrophage polarization. In this study, we investigated the effects of a novel, highly selective and potent BTK inhibitor, BI-BTK-1, in an inducible model of LN in which mice receive nephrotoxic serum (NTS) containing anti-glomerular antibodies. Mice were treated once daily with vehicle alone or BI-BTK-1, either prophylactically or therapeutically. When compared with control treated mice, NTS-challenged mice treated prophylactically with BI-BTK-1 exhibited significantly attenuated kidney disease, which was dose dependent. BI-BTK-1 treatment resulted in decreased infiltrating IBA-1+ cells, as well as C3 deposition within the kidney. RT-PCR on whole kidney RNA and serum profiling indicated that BTK inhibition significantly decreased levels of LN-relevant inflammatory cytokines and chemokines. Renal RNA expression profiling by RNA-seq revealed that BI-BTK-1 dramatically modulated pathways related to inflammation and glomerular injury. Importantly, when administered therapeutically, BI-BTK-1 reversed established proteinuria and improved renal histopathology. Our results highlight the important role for BTK in the pathogenesis of immune complex-mediated nephritis, and BTK inhibition as a promising therapeutic target for LN.

Systemic lupus erythematosus (SLE) is an autoimmune disease characterized by autoantibody production and systemic inflammation which culminates in various end organ pathologies. Kidney involvement, known as lupus nephritis (LN), affects upwards of 60% of SLE patients, and adds considerable morbidity and mortality to the disease^1^.

Current therapies for lupus nephritis (LN) consist mainly of non-specific immunosuppression which can be associated with dangerous side effects, yet often fail at producing long term remission. Further research efforts into the pathogenesis of LN and new therapeutic targets are necessary to improve patient care and the long term prognosis^2^.

B cells and macrophages are believed to be important in the pathogenesis of LN^3^. Autoantibody complexes deposited in the kidneys can activate complement cascades and Fc receptors on local and infiltrating cells, thus leading to renal injury^4^. Furthermore, activated renal macrophages are markers for disease onset and remission^5^, and depletion of macrophages ameliorates disease – indicating their importance in LN^6^,^7^.

Bruton’s tyrosine kinase (BTK), a member of the Tec family of non-receptor tyrosine kinases, is essential for intracellular signaling in B cells and myeloid lineages. The role of BTK in BCR signaling is exemplified by the impaired B cell development and function observed in human X-linked agammaglobulinemia and X-linked immunodeficiency mice, which harbor specific BTK mutations^8^,^9^. BTK is also needed for FcR signaling which mediates immune complex (IC) activation of myeloid cell types such as monocytes and macrophages^10^. Finally, BTK expression is significantly upregulated in LN patients^11^. Thus, targeting BTK may be a promising therapeutic target in LN, as it affects both B cell and macrophage function.

In this study we utilized a classic experimental model known as nephrotoxic serum nephritis (NTN), that relies on the passive transfer of pre-formed nephrotoxic antibodies into mice to induce IC-mediated nephritis. The resulting proliferative glomerulonephritis is characterized by IC deposition, complement activation, and immune cell infiltration. Since NTN is highly similar, histologically and mechanistically, to the glomerulonephritis seen in SLE, it is commonly used as a model for this particular lupus manifestation^12^.

We investigated the role of a novel, highly selective, and potent BTK inhibitor, BI-BTK-1 (Patent publication WO2014025976), in NTN. We implemented prophylactic treatment to investigate the role of BTK in the pathogenesis of antibody mediated nephritis. Additional studies demonstrated the significant therapeutic effect of BI-BTK-1 in NTN, highlighting the potential of BI-BTK-1 as a treatment option for LN and other antibody mediated glomerulopathies.

## Results

### BI-BTK-1 is a Selective Potent Inhibitor of BTK

**Figure 1:**
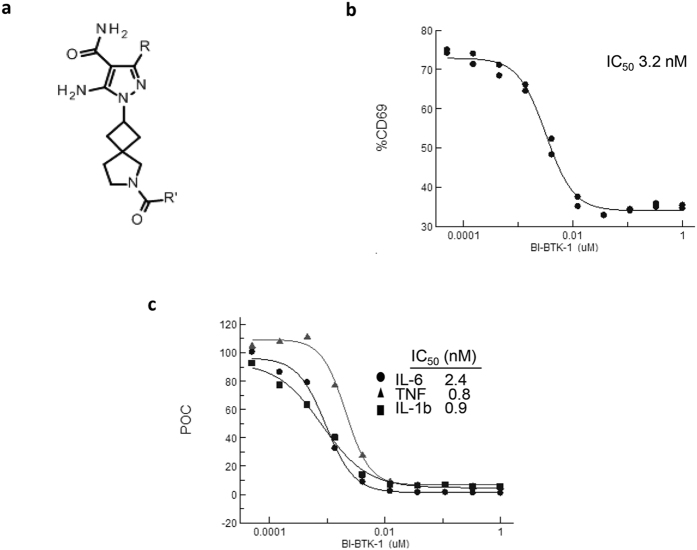
Cellular activity of BI-BTK-1. (**a**) Chemical structure of BI-BTK-1. (**b**) Inhibition of αIgD stimulated CD69 expression on primary human CD19+ B cells isolated from PBMC. Percentage of CD69+ cells as determined by FACS is presented. (**c**) BI-BTK-1 inhibition of anti-human serum albumin immune complex stimulated IL-6 (⦁), TNF (▴) and IL-1b (▪) secretion in human CD14+ monocytes. POC, Percentage of control. Panels (**b**,**c**) each display representative data from a single donor.

BI-BTK-1 is a potent, small molecule inhibitor of BTK (Fig. 1a) that forms an irreversible covalent bond between the electrophile present in R‘ and cysteine 481 located near the ATP binding pocket of the kinase domain, as determined by co-crystallization and mass spectrometry (not shown). Due to its irreversible binding, BI-BTK-1 displayed time dependent (Kinact/Ki = 85,000 1/M sec) and potent (IC50 = 0.9 nM) inhibition of BTK enzymatic activity (Table 1). BI-BTK-1 potently inhibited BCR stimulated B cell activation, as measured by CD69 expression in primary human CD19+ B cells isolated from PBMCs (Fig. 1b) and human whole blood (Supplemental Fig. 1), as well as the secretion of cytokines from IC stimulated human CD14+ monocytes (Fig. 1c). BI-BTK-1 (up to 10 μM) had no effect on CD3/CD28 stimulated T cell activation (not shown). Results of molecular and cellular testing of BI-BTK-1 are summarized in Table 1. Testing of BI-BTK-1 in a kinase selectivity panel revealed >80% inhibition at 3 μM for only 8/282 kinases tested (Supplemental Table 1).

### BI-BTK-1 Abrogates Kidney Disease in NTN

**Figure 2:**
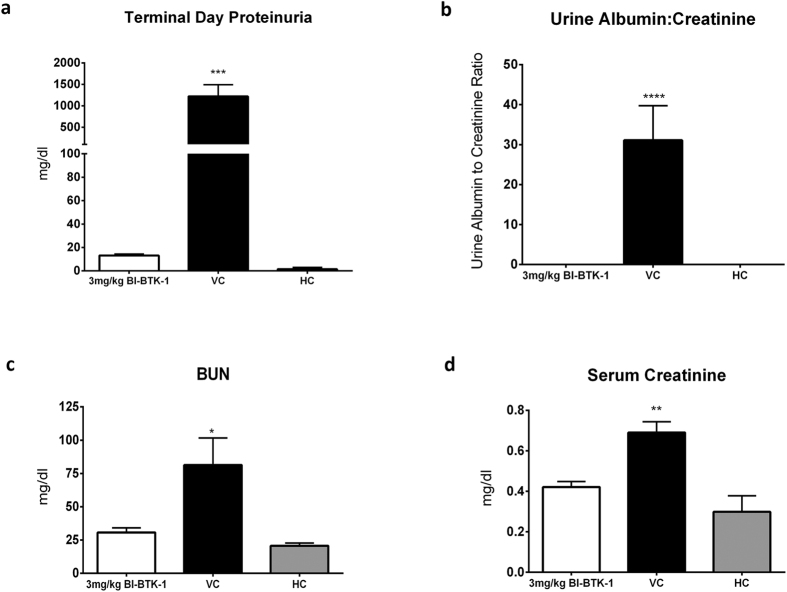
Renal function in NTS-challenged, BI-BTK-1 treated mice. Proteinuria levels were measured at terminal day 11 via uristix (**a**). Urine albumin levels were measured by ELISA and then normalized to urinary creatinine levels to adjust for urinary output (**b**). Renal function was analyzed by measuring serum BUN (**c**) and creatinine (**d**) by ELISA. Shown here are results from one experiment (BI-BTK-1 treated, n = 8; VC, n = 8; HC, n = 5). Asterisks represent VC being statistically different than each group (*p < 0.05, **p < 0.01, ***p < 0.001, ****p < 0.0001).

129 sv/J mice were injected with nephrotoxic serum to induce antibody mediated glomerulonephritis mimicking spontaneous LN. Vehicle treated, sick control mice (VC) began developing significantly increased levels of proteinuria (as assessed by uristix) starting on day 7 and peaking on day 11. However, mice treated with BI-BTK-1 at 3 mg/kg starting on day 4 did not develop proteinuria and were never significantly different than HC mice (Fig. 2a). Urine albumin:creatinine levels, as measured by ELISA, confirmed these findings (Fig. 2b). Further, serum creatinine and BUN were measured to assess kidney function. As seen with the analysis of proteinuria, mice treated with BI-BTK-1 were protected from developing the kidney dysfunction appearing in the VC group (Fig. 2c,d).

**Figure 3:**
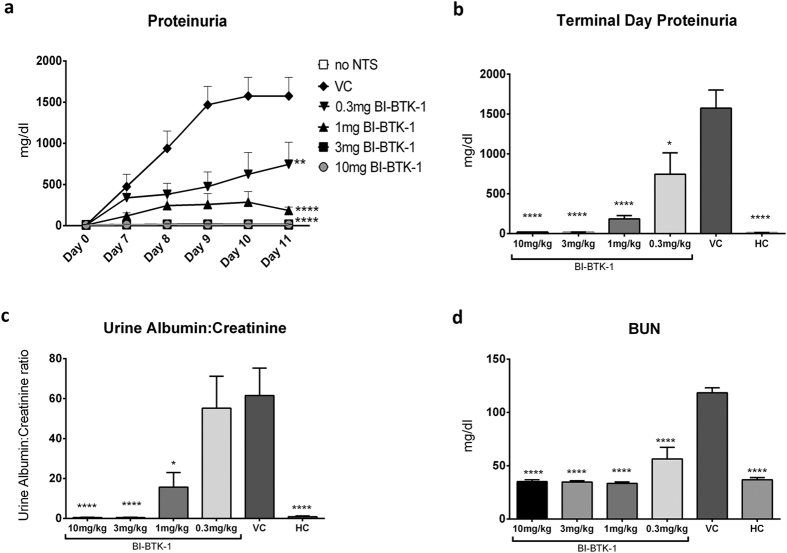
Dose response to BI-BTK-1. Proteinuria levels were measured on the designated days by uristix (**a**). By day 11, BI-BTK-1 treated mice showed a dose responsive level of protection from development of the high levels of proteinuria seen in VC mice (**b**), as confirmed by urine albumin/creatinine ratios measured by ELISA on the day of sacrifice (**c**). Kidney function was also preserved in treated mice, as assessed by BUN levels (**d**). Shown here are results from one experiment (10 mg/kg BI-BTK-1 treated, n = 8; 3 mg/kg BI-BTK-1, n = 8; 1 mg/kg BI-BTK-1, n = 8; 0.3 mg/kg BI-BTK-1, n = 8; VC, n = 8; HC, n = 5). Asterisks represent a significant difference compared to VC (*p < 0.05, **p < 0.01, ****p < 0.0001).

Since 3 mg/kg BI-BTK-1 provided meaningful protection from the nephrotoxic effects of the transferred antibodies, we were interested in determining the effect of various doses of this compound. Consequently, we implemented a dose response experiment testing 0.3, 1, 3, and 10 mg/kg of BI-BTK-1. As seen in Fig. 3, BI-BTK-1 ameliorated nephritis in a dose responsive manner. As in the first experiment, 3 mg/kg of BI-BTK-1 given from day 4 fully prevented proteinuria following the nephrotoxic challenge, and normalized terminal urinary albumin:creatinine and BUN levels. A dose of 10 mg/kg was similarly effective. The dose of 1 mg/kg provided intermediate protection, while 0.3 mg/kg was significantly less effective. Overall, this experiment revealed 3 mg/kg as the optimal dose, as it was the lowest amount of BI-BTK-1 given that still provided maximum functional protection.

### BTK Inhibition Prevents Renal Damage

**Figure 4:**
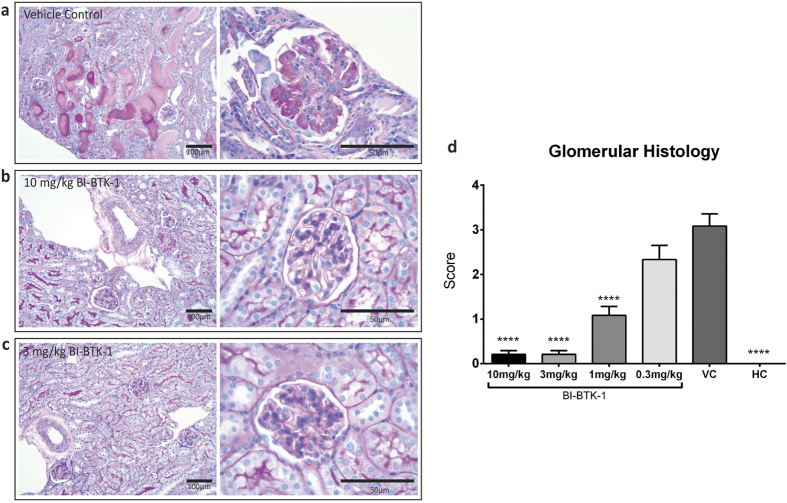
Renal histopathology. Panel (**a**) (left) displays a representative image (200×) of renal histology in the VC treated mice displaying tubular dilatation and tubular casts, as well as notably hypercellular glomeruli. Increased magnification (**a**) right (600×)) reveals large amounts of immune deposition, global proliferation, and an incipient crescent. In contrast, panels (**b**,**c**) show normal renal architecture in BI-BTK-1 treated mice (10 mg/kg and 3 mg/kg respectively). Glomerular histology scores are shown in panel (**d**). Shown here are results from one experiment (10 mg/kg BI-BTK-1 treated, n = 8; 3 mg/kg BI-BTK-1, n = 8; 1 mg/kg BI-BTK-1, n = 8; 0.3 mg/kg BI-BTK-1, n = 8; VC, n = 8; HC, n = 5). Asterisks represent a significant difference compared to VC (****p < 0.0001).

VC mice displayed severe histological glomerulonephritis (Fig. 4a), with high glomerular (Fig. 4d) and tubular (Supplemental Fig. 2) disease scores. Consistent with the biochemical measures of kidney function, the renal histology results showed a significant dose response to BI-BTK-1. Mice treated with BI-BTK-1 at 10 mg/kg or 3 mg/kg showed virtual amelioration of disease, while doses of 1 mg/kg and 0.3 mg/kg showed intermediate protection (Fig. 4b–e; Supplemental Fig. 2).

### BTK Inhibition Does Not Interfere With Induction of NTN

**Figure 5:**
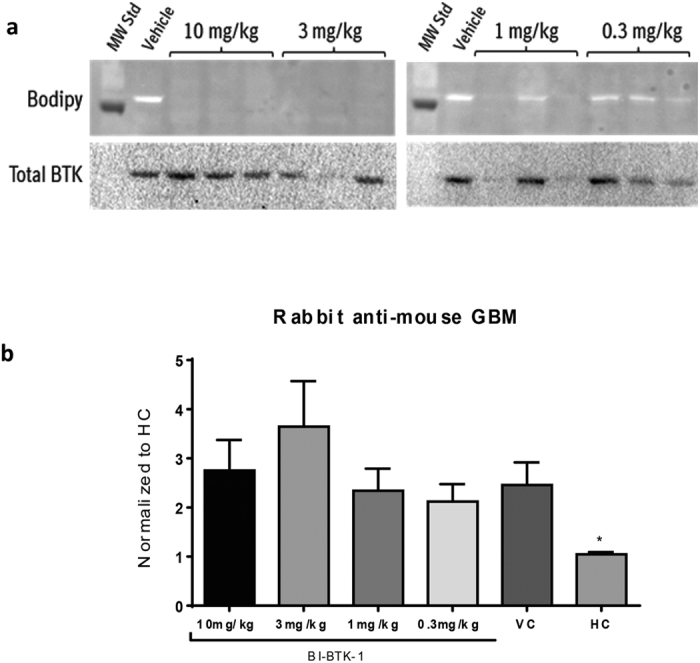
Disease induction checkpoints. (**a**) Effect of BI-BTK-1 on % BTK target occupancy. (Top) Fluorescence scanning of splenocyte lysates that were incubated with a bodipy-labeled irreversible BTK inhibitor, followed by SDS-PAGE and transfer to nitrocellulose. (Bottom) Western blot detection of total BTK protein in splenocyte lysates used in the top panel. Mean % ± S.D BTK TO max was 95 ± 3, 89 ± 7, 52 ± 28, and 33 ± 14 for 10, 3, 1 and 0.3 mg/kg groups (n = 3/group), respectively. The corresponding mean ± SD (nM) plasma concentrations of BI-BTK-1 1 hour post dosing were 193.3 ± 80.9, 79.3 ± 40.4, 9.7 ± 4.9 and 4 ± 4.6 for 10, 3, 1 and 0.3 mg/kg groups (n = 3/group), respectively. BI-BTK-1 did not interfere with the induction of disease, as assessed by rabbit anti-mouse GBM levels (**b**) in the terminal serum. Shown here are results from one experiment (10 mg/kg BI-BTK-1 treated, n = 8; 3 mg/kg BI-BTK-1, n = 8; 1 mg/kg BI-BTK-1, n = 8; 0.3 mg/kg BI-BTK-1, n = 8; VC, n = 8; HC, n = 5). Asterisks represent a significant difference of HC compared to all other groups (*p < 0.05).

To confirm BTK target occupancy (TO) after treatment with BI-BTK-1, the percentage of BTK TO was determined via incubation of isolated splenocytes with a fluorescent bodipy-labeled BTK covalent warhead inhibitor probe, followed by Western blot detection of total BTK protein (Fig. 5a). Treatment with BI-BTK-1 resulted in a dose dependent decrease of bodipy-labeled BTK, indicating target engagement of this covalent, irreversible BTK inhibitor.

The basis of the NTN model is kidney deposition of ICs, consisting of the antibodies resulting from the initial immunization cross-linking the glomerular-deposited nephrotoxic antibodies passively transferred on day 5. To ensure that the amelioration of disease was not due to the BTK inhibition altering either of these two antibody populations, we measured their serum levels by ELISA. There were no significant differences between any of the groups in levels of mouse anti-rabbit IgG, indicating that BTK inhibition did not interfere with the antibody response elicited on day 0 (Supplemental Fig. 3). Thus, differences in cross-linking of the deposited rabbit anti-mouse GBM antibodies did not contribute to the therapeutic effect exerted by BI-BTK-1.

All groups save for the HC group received nephrotoxic antibodies. Each of these five groups had significantly higher levels of rabbit anti-mouse GBM antibodies (with no significant difference between them) than the HC group, which was injected with PBS rather than NTS (Fig. 5b).

### BTK Inhibition Reduces Kidney and Serum Cytokines

Induction of NTN results in marked increases in inflammatory cytokines both within the kidney and systemically. To explore the mechanisms by which BI-BTK-1 exerts its renoprotective effect and assess the effect of BTK inhibition on cytokine expression, RT-PCR was run on whole kidney RNA extracts and Luminex was performed on terminal serum.

**Figure 6:**
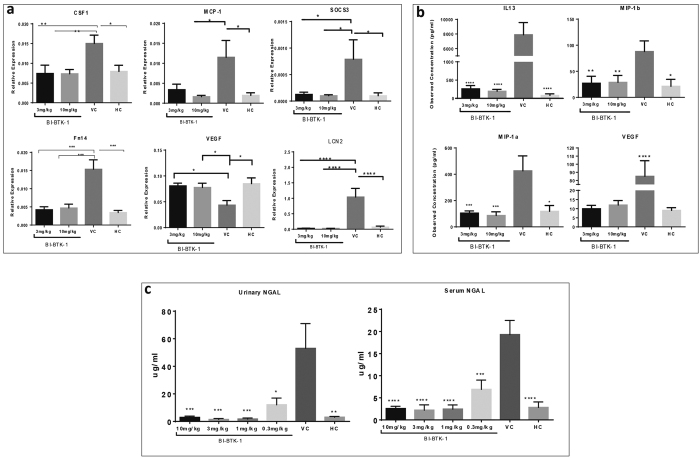
Markers of kidney disease. Kidney (**a**) and serum (**b**) expression of cytokines associated with kidney disease were assessed by RT-PCR and luminex, respectively. Additionally, NGAL levels were assessed in both the terminal urine and serum by ELISA (**c**). Asterisks represent a significant difference of VC compared to all other groups (*p < 0.05, **p < 0.01, ***p < 0.001, ****p < 0.0001).

Within the kidney, BTK inhibition reduced the mRNA expression of LCN2 (encoding for NGAL) and other mediators associated with LN, including (but not limited to) MCP-1, Fn14, and CSF-1 (Fig. 6a). Interestingly, many of these genes are associated with macrophage function, including MCP-1 which is vital in attracting macrophages to the inflamed kidney, and CSF-1 which is important in macrophage recruitment, activation, and proliferation^13^,^14^. Furthermore, SOCS3, which is upregulated in glomerular infiltrating inflammatory (M1) macrophages during nephritis^15^, was downregulated by BTK inhibition. This data is consistent with the observed benefit of BTK inhibition being mediated by interference with macrophage effector function.

Similarly, BTK inhibition reduced inflammatory molecules systemically, with IL-13, VEGF, MIP-1α, and MIP-1β significantly decreased in the serum of BI-BTK-1 treated mice (Fig. 6b). In order to understand the changes in the expression of these proteins in the kidney, qPCR was performed as well. VEGF was decreased in the VC group and treatment with BI-BTK-1 inhibitor normalized (increased) its expression (Fig. 6a). There was no expression found of IL-13, and no significant differences in MIP-1α and MIP-1β were observed (not shown). Furthermore, NGAL was significantly reduced both in the urine and serum of BI-BTK-1 treated mice (Fig. 6c).

**Figure 7:**
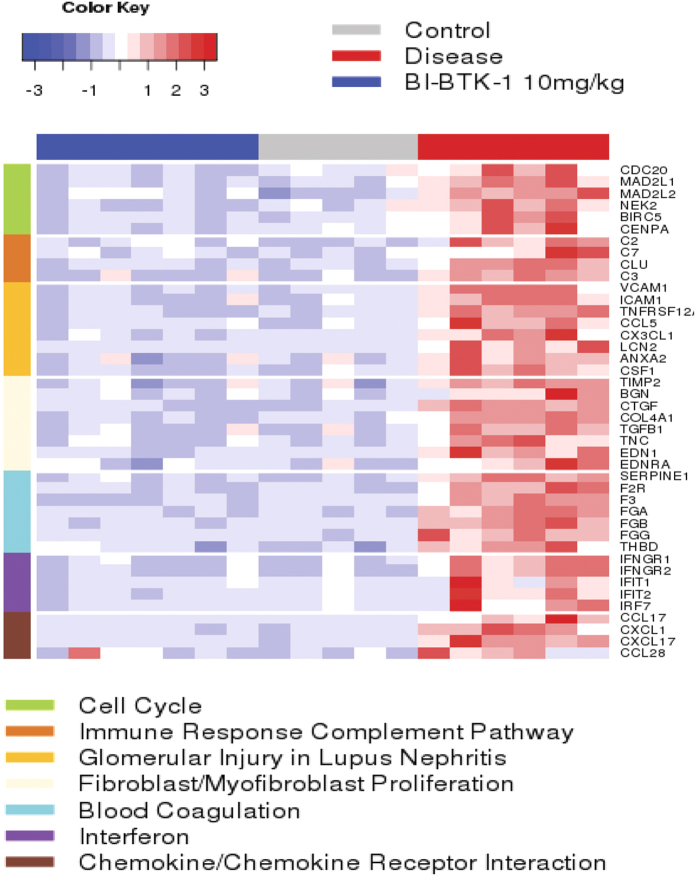
RNA seq analysis. Differentially expressed genes in the kidneys of treated mice with FDR < 0.05 and a fold change cutoff of 1.5 were used to query pathways databases including Ingenuity Pathway Analysis and the MetaCore database. Significantly modulated genes from top ranked pathways (by FDR) are listed in the RNAseq heatmap using z score of normalized gene expression level. The results were from one experiment (10 mg/kg BI-BTK-1 treated, n = 7; VC, n = 6; HC, n = 5).

Many pathways related to inflammation and/or LN are induced in NTN-challenged mice. RNA Seq analysis showed >500 genes modulated by treatment with 10 mg/kg BI-BTK-1 (using q value <0.05, fold change (DEseq2) cutoff at 1.5). The heatmap (Fig. 7) and Supplemental Table 2 show the top NTN-induced pathways, and how BTK treatment modulated these genes. Besides the effects on LCN2, VCAM-1, and CSF-1 described above, RNA seq demonstrated that BI-BTK-1 significantly modulated the expression of many additional genes including those related to activation of the complement, coagulation, and IFN pathways. RNA seq in the 3 mg/kg BI-BTK-1 treated group showed similar trends (not shown).

### BTK Inhibition Preserves the Splenic Reservoir of Monocytes and Decreases Kidney

#### **IBA-1+ Cells**

**Figure 8:**
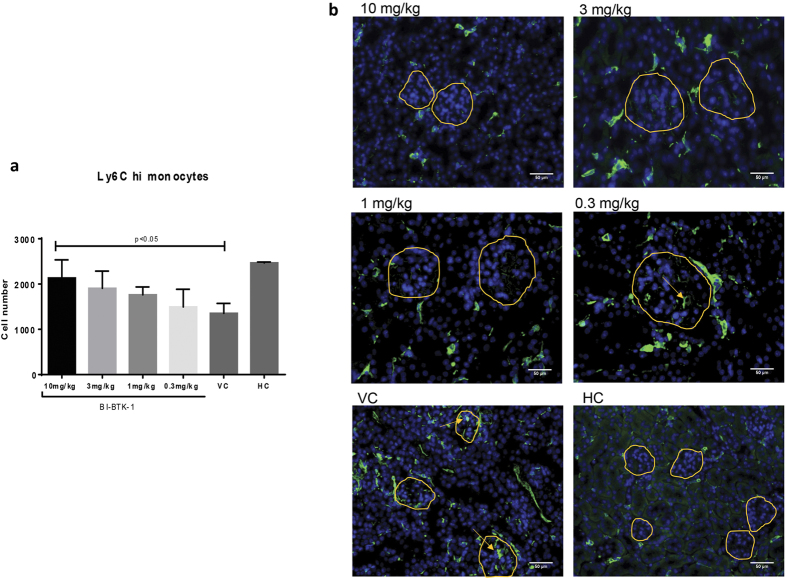
Monocyte and macrophage trafficking. Spleens from perfused mice at sacrifice were processed into a single cell suspension and stained for splenic monocytes to assess the effect of BI-BTK-1 on monocyte trafficking from the splenic reservoir. Decreased numbers, as seen in the VC group, are consistent with recruitment of these monocytes to sites of inflammation. 10 mg/kg BI-BTK-1 treated mice had significantly more monocytes than VC and were comparable to HC (**a**). Additionally, IBA-1 staining was performed to assess peri- and intraglomerular macrophage accumulation (**b**). Shown here are results from one experiment (10 mg/kg BI-BTK-1 treated, n = 8; 3 mg/kg BI-BTK-1, n = 8; 1 mg/kg BI-BTK-1, n = 8; 0.3 mg/kg BI-BTK-1, n = 8; VC, n = 8; HC, n = 5).

The spleen houses a monocyte reservoir which can be rapidly mobilized to the site of inflammation^16^. In NTN (Fig. 8), VC mice show significantly depleted monocytes within the spleen. Interestingly, mice treated with BI-BTK-1 at 10 mg/kg had a monocyte pool within their spleens comparable to HC mice, presumably since the chemotactic signal was significantly attenuated in the kidney. A similar trend was seen as well in mice treated at a dose of 3 mg/kg (p = 0.09) (Fig. 8a).

In order to investigate the accumulation of macrophages within the kidney, sections were stained with IBA-1 to assess peri- and intraglomerular accumulation. HC mice had normal interstitial macrophage populations but lacked the periglomerular and intraglomerular macrophages noted in the VC mice. Mice dosed with 0.3 mg/kg of BI-BTK-1 also showed an accumulation of intra- and periglomerular macrophages, while mice given the higher doses were protected from this accumulation and appear much more similar to HC mice (Fig. 8b).

### BTK Inhibition Reduces Complement Accumulation in the Kidney

**Figure 9:**
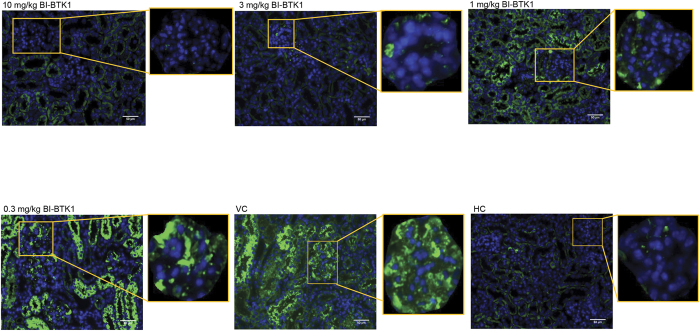
Complement deposition in the kidney. Kidney C3 deposition was inversely related to the dose of BI-BTK-1 given to the mice, with the amount of deposited C3 in the 10 mg/kg and 3 mg/kg groups appearing very similar to HC mice. Shown here are results from one experiment (10 mg/kg BI-BTK-1 treated, n = 8; 3 mg/kg BI-BTK-1, n = 8; 1 mg/kg BI-BTK-1, n = 8; 0.3 mg/kg BI-BTK-1, n = 8; VC, n = 8; HC, n = 5).

Kidney complement activation and deposition occurs in NTN mice following IC formation. To assess the effect of BTK inhibition on kidney complement deposition, sections were stained for C3. As seen in Fig. 9, the amount of complement deposition in the kidney was inversely related to the dose of BI-BTK-1, and at higher doses was significantly decreased compared to VC.

### BI-BTK-1 Reverses Established Nephritis in NTN

**Figure 10:**
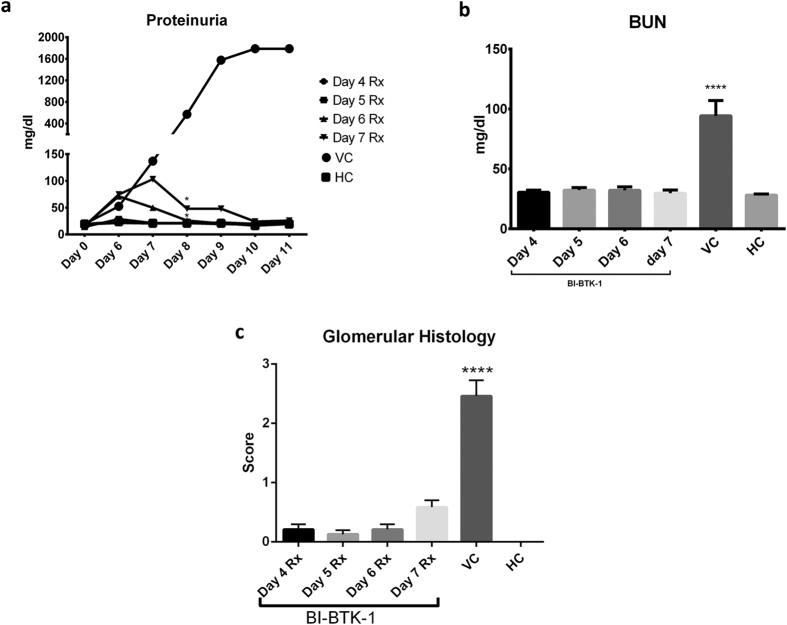
Delayed treatment of NTN with BI-BTK-1. BI-BTK-1 (3 mg/kg) treatment was delayed from day 4 to subsequent days (5, 6, and 7) to assess the therapeutic potential in established disease. Treatment was able to stop the progression of nephritis within a day of starting treatment (**a**). By day 8 and continuing until the day of sacrifice, the proteinuria levels for every BI-BTK-1 treatment group was significantly different than the VC group. At sacrifice, all treated groups had normal BUN levels despite a later start to the treatment (**b**). Glomerular histology confirmed the beneficial effect of BI-BTK-1 treatment (**c**). (day 4 start, n = 8; day 5 start, n = 8; day 6 start, n = 8; day 7 start, n = 8; VC, n = 8; HC, n = 5). Asterisks represent a significant difference of VC compared to all other groups (*p < 0.05, ****p < 0.0001).

To assess the potential of BI-BTK-1 as a treatment for antibody-mediated nephritis, treatment was staggered to start on different days to assess the effect on established disease. Interestingly, treatment with BI-BTK-1 was able to not only prevent the development of proteinuria, but also reverse it in mice with early nephritis (Fig. 10a). Further, BUN levels in all treated groups were comparable to HC mice (Fig. 10b). Promising results were particularly noted when BI-BTK-1 treatment was started as late as day 7. As can be seen in Fig. 10a, these mice began exhibiting elevated levels of proteinuria comparable to the VC mice. However, one day after beginning treatment, proteinuria decreased to levels comparable to HC mice. Analysis of renal histology confirmed the efficacy of the delayed treatment (Fig. 10c; Supplemental Fig. 4). These results point to the ability of BTK inhibition to not only prevent onset of nephritis following subsequent exposure to pathogenic antibodies but also to reverse already established disease, at least in its early stages.

## Discussion

Current treatment options for LN are limited and not ideal due to their incomplete efficacy, cytotoxicity, and immunosuppressive effects. Patients would benefit greatly from more targeted interventions, and BTK is a specific target that holds therapeutic promise.

We showed here that prophylactic BTK inhibition ameliorates NTN-mediated kidney disease. Specifically, treated mice were protected in a dose dependent manner from developing the abnormally elevated proteinuria and renal dysfunction seen in control treated mice. Furthermore, analysis of renal histopathology confirmed the complete renal protection provided by BTK inhibition. Finally, BTK inhibition did not interfere with the induction of the disease model.

Further analysis of cytokines both in the kidneys and serum revealed that BTK inhibition abrogated the upregulated expression of those inflammatory mediators seen in sick control mice. Specifically, many of these cytokines are related to macrophage function – indicating that BTK inhibition may be mechanistically abrogating disease due to its interference with macrophages, which contribute to pathogenesis. We found further evidence pointing to interference with macrophage effector function when assessing the splenic monocyte pool, which was depleted in sick VC mice, but maintained at higher doses of BI-BTK-1.

NGAL is a biomarker of LN^17^, and more broadly kidney disease, with elevated levels serving as a non-invasive marker of the extent of renal damage. The role of NGAL goes beyond that of a biomarker, however, since NGAL is known to play a critical role in nephritis by promoting inflammation and apoptosis within the kidney^18^. We saw dramatic increases of NGAL within the kidney, urine, and serum of VC treated mice; treatment with BI-BTK-1, however, maintained NGAL at levels comparable to HC mice, supporting the promising potential of this drug in ameliorating renal disease.

Several studies have previously looked at the potential role of BTK inhibition in LN. Honigberg *et al*. studied the effect of the BTK inhibitor ibrutinib in MRL/lpr lupus-prone mice starting pre-disease (8 weeks). Mice were treated for 12 weeks with three different doses, with significant improvement in proteinuria. Histopathological analysis revealed an overall improvement at the two higher doses, but no significant differences in glomerular histology^19^. Hutchenson *et al*. evaluated the same BTK inhibitor in the SLE1.3 lupus model at an estimated dose of 30 mg/kg. This study specifically looked at the effect of BTK inhibition on B cell function, noting decreased humoral immunity which dampened LN. Specifically, treated mice displayed delayed production of autoantibodies, decreased splenomegaly, and attenuated nephritis^20^.

Another study by Mina-Osorio *et al*. investigated a chow form of a BTK inhibitor, RN486, in NZB/W F1 mice, also at an estimated dose of 30 mg/kg. Mice began treatment at 32 weeks of age, once they displayed mild proteinuria. The treatment halted LN disease progression, and lowered proteinuria to below baseline levels^21^. Similarly, Rankin *et al*. reported that prophylactic treatment with PF-06250112 in NZB/W F1 mice improved nephritis^22^. However, any salutatory effect on spontaneous disease in these lupus-prone strains could have been mediated via a decrease in autoantibody titers, rather than a local effect on kidney inflammation. Moreover, although Rankin *et al*. also reported a beneficial effect of PF-06250112 in NTN, the degree of proteinuria was not measured quantitatively and the mechanism was not further elaborated.

Our study highlights a potent and selective BTK inhibitor that not only works prophylactically but, more importantly, demonstrates efficacy in a therapeutic setting. While this model does not allow us to explore the effects on B cells, the published data with BTK inhibitors on B cells, and our *in vitro* data, suggests that BTK inhibition can also affect B cell autoimmunity. In addition, the data from this study shows that by inhibiting IC mediated activation of myeloid cells there is an additional benefit of inhibiting many inflammatory pathways/genes relevant to disease pathogenesis and progression. Inhibition of BTK attenuates key pathways of kidney damage such as complement activation, leukocyte chemotaxis, interferon inducible genes, and other pathways/genes associated with LN. Furthermore, the down modulation of IFN regulated genes in BTK treated mice found in the RNA seq analysis is consistent with BTK regulation of IC triggered monocyte and IFN pathway activation^21^. Whether the source of IFN is from T_H_1 cells that infiltrate the kidneys or from kidney resident cells, we found that IFN regulated genes which are associated with disease pathogenesis are modulated by BTK inhibition. Furthermore, from a clinical standpoint, delayed treatment not only halts disease progression but also reverses established disease, as seen by the decrease in proteinuria, improved renal histopathology, and normalization of BUN at time of sacrifice. These data, combined with the specificity of our compound, provides confidence in the efficacy of the novel inhibitor reported in this study.

BI-BTK-1 also holds promise for clinical translation, as BTK inhibitors are already in human trials for non-renal indications without any common severe side effects requiring the discontinuation of therapy. In a summary of the safety and efficacy of ibrutinib for chronic lymphocytic leukemia the majority of the reported side effects (upper respiratory tract infection, fatigue, diarrhea) were grade 1 or 2, thus allowing patients to undergo extended treatment with the drug^23^. Highlighting the potential for an improved therapeutic ratio of the novel BTK inhibitor described herein, biochemical profiling here demonstrated that many of the kinases inhibited by ibrutinib at pharmacological concentrations are not inhibited by BI-BTK-1. Specifically, BI-BTK-1 does not significantly inhibit EGFR (associated with adverse events such as rash and diarrhea) or ITK (critical for NK function).

We believe that our results highlight a new BTK inhibitor with improved selectivity over existing BTK inhibitors and promising therapeutic potential for autoimmune renal diseases. Furthermore, our study reiterates the importance of BTK in the development of LN, and highlights this enzyme as an important therapeutic target. Although the role of BTK inhibition in nephritis was considered in previous studies, the rapid onset of action and potency of BI-BTK1 in a hyperacute and severe model, its efficacy in reversing established disease, its high selectivity over other BTK inhibitors, and the dissection of the mechanisms of protection are novel features of this particular inhibitor. Finally, our results uniquely indicate that BTK inhibition within macrophages may be a key mechanistic target and potentially promising therapeutic approach in the future treatment of LN.

## Methods

### Mice

129/SvJ mice were purchased from The Jackson Laboratory and housed at the Albert Einstein College of Medicine animal facility (Bronx, NY). The Institutional Animal Care Committee approved all animal studies. The experiments performed in this study were carried out in accordance with the approved guidelines.

### Nephrotoxic Serum Transfer

Nephrotoxic serum nephritis was induced as described^7^. Briefly, blood and urine were collected for baseline measurements from 10 week old, female 129sv/J mice, which were then immunized with CFA and rabbit IgG via intraperitoneal injection. All days referred to below will consider the date of initial immunization with rabbit IgG as day 0, with subsequent time points in reference to that baseline. On day 5, mice were intravenously injected with either nephrotoxic serum (NTS) or PBS. Mice were monitored daily for proteinuria development via uristix (Siemens Healthcare Diagnostic, Tarrytown, NY) starting on day 7.

Several groups of mice were used to explore the effect of the BTK inhibitor, BI-BTK-1, to prevent the onset of disease or attenuate its severity. Two groups (BI-BTK-1 and vehicle control (VC); n = 8 in each) were immunized on day 0 and given NTS on day 5. The BTK inhibitor was given at a dose of 3 mg/kg suspended in vehicle (0.5% Natrosol, 0.015% Tween 80) via oral gavage daily starting on day 4, while the VC group was given a control gavage of the vehicle daily, and thus acted as a sick control group. The last group was a healthy control (HC) group which was immunized on day 0 but injected with PBS on day 5 (n = 5).

Separate experiments were performed with BI-BTK-1 which varied either the daily therapeutic dose (0.3 mg/kg, 1 mg/kg, 3 mg/kg, or 10 mg/kg) or the day of initial treatment (day 4, day 5, day 6, and day 7). This allowed us to confirm the initial results in independent cohorts, and assess both the dose response and therapeutic potential of the drug.

### Assessment of Proteinuria and Renal Damage

Uristix test strips were used to assess proteinuria levels daily based upon the color change. Intermediate color changes were assigned the midrange proteinuria value. Albumin levels were measured with the Albumin ELISA Quantification Set (Bethyl Laboratories, Montgomery, TX) following the manufacturer’s protocol. Serum and urinary creatinine were determined by QuantiChrom Creatinine Assay kit (BioAssay Systems, Hayward, CA) and blood urea nitrogen (BUN) levels were measured via DIUR 500 kit (BioAssays).

### Renal Histopathology

Kidney sections were deparaffinized and stained with hematoxylin and eosin (H&E) and periodic acid Schiff (PAS) by the Histology and Comparative Pathology Core at the Albert Einstein College of Medicine. Kidney sections were then scored by an experienced nephropathologist (L.H.) who was blinded to the experimental groups. Scoring was assigned as described^7^. Briefly, sections were assessed for glomerular deposits, endocapillary proliferation, glomerular crescent formation, interstitial inflammation, and tubular casts and dilatation. Each category was then assigned a score of 0–4, where 4 is severe disease and 0 is no disease or normal appearing histology. Scores for glomerular deposits, endocapillary proliferation, and glomerular crescent formation were averaged to obtain a score for glomerular histology; scores for interstitial inflammation and tubular casts and dilatation were averaged to obtain a score for tubular histology.

### Mouse Anti-Rabbit IgG and Rabbit Anti-Mouse Glomerular Basement Membrane (GBM) Antibodies

Mouse anti-rabbit IgG and rabbit anti-mouse GBM antibody serum titers were measured by ELISA, as previously described^24^.

### BTK Target Occupancy Determination

For assessment of BTK target occupancy, spleens were harvested two hours after the final oral dose of BI-BTK-1. Isolated splenocytes were rinsed in media (RPMI 1640 w/o L-Glutamine + 1% FBS), pelleted and resuspended in red blood cell lysing buffer (Sigma-Aldrich) for 10 minutes. Upon resuspension in media, splenocytes were incubated with a bodipy-labeled covalent probe (5 μM final) for 1 hour at 37 °C. After freeze/thaw lysis and centrifugation to remove insoluble material, splenocyte extracts were analyzed by SDS-PAGE. BTK target occupancy was calculated by measurement of the fluorescent signal provided by bound bodipy-covalent probe using a ChemiDoc Imaging System (Bio-Rad, ex, 615–645 nm; em, 670–724 nm) combined with correction for the total amount of BTK loaded as measured by western blot analysis (Cell Signaling).

### RNA Isolation, cDNA Synthesis, Real time PCR, and RNA Seq

RNA was isolated from snap frozen kidneys by homogenization in Trizol using a Retsch MM300 Tissue Lyser. Chloroform was added and the aqueous phase was processed using the Agencourt RNAdvanced tissue kit that was modified for automation on a Biomek FXp from Beckman. For Taqman analysis, reverse transcription was performed using the TaqMan Reverse Transcription Reagents Kit (Applied Biosystems). The resultant cDNA was used in a ViiA 7 Real-Time PCR system (Applied Biosystems) using mouse specific probes from Applied Biosystems. Statistical analysis was done by ANOVA, followed by Dennett’s T-test in GraphPad Prism.

RNA seq was performed commercially by Labcorp (Seattle, WA). Single end RNA-seq reads were mapped with STAR 2.4. Absolute read counts/gene were generated using Subread’s feature Counts program v1.4.6 and the gtf annotation file for mouse mm10 reference from UCSC. Differential expression analysis was performed with the DESeq2 package. Functional annotation analysis for the differentially expressed genes was performed using pathway analysis tools including Ingenuity Pathway Analysis (QIAGEN) and MetaBase (Thomson Reuters).

### BTK Binding Assays

The affinity of BI BTK-1 was measured with a time resolved-FRET LanthaScreen binding assay following the manufacturer’s instructions (Invitrogen) using a PerkinElmer Viewlux (ex. 340 nM; em. 615/665 nM). For determination of K_inact_/K_i_ the binding assay was monitored every 60 seconds over 60 minutes (M^−1^ S^−1^), as described (Singh 1997)^25^.

### Cellular and Whole Blood Assays

Primary CD19+ cells were purified from human peripheral blood mononuclear cells (AllCells) and negatively selected by magnetic separation with >97% purity (Stemcell Technologies). After incubation with BI-BTK-1 or vehicle alone (1% DMSO) for 1 hour, cells were stimulated with 12.5 μg/ml goat F(ab’)2 anti-human IgD (Southern Biotech) for 18–24 hours. After staining for APC-CD19 and PE-CD69 (BD Bioscience), B cells were analyzed by flow cytometry using a BD LSRII Flow Cytometer. Viable cells were gated, and % CD69+ was determined using FlowJo software. Whole blood samples obtained from healthy volunteers drawn in sodium heparin tubes (BD Biosciences) were treated with BI-BTK-1 or vehicle control (1% DMSO) for 1 hour followed by stimulation as described above. Samples were stained in blocking buffer (PBS, 10% human serum, 0.1% sodium azide) for APC-CD19 and PE-CD69. After red blood cell lysis (BD Biosciences), flow cytometry was performed and % CD69+ CD19+ B cells were determined using FlowJo software.

To determine the potency of BI-BTK-1 in inhibiting IC mediated activation of monocytes, CD14+ cells were purified from healthy frozen peripheral blood mononuclear cells (AllCells) and negatively selected by magnetic separation with >97% purity (Stemcell Technologies). Cells were plated at a concentration of 2 × 10^5^/well in RPMI media containing 10% FBS and treated with BI-BTK-1 or vehicle alone (1% DMSO) for 1 hour. Cells were transferred in media to a microtiter plate containing immobilized human serum albumin immune complexes prepared as described previously^21^. After incubation for 18–24 hours, the amount of IL-1β, IL-6, and TNF in the supernatant was measured via ELISA (Meso Scale Discovery).

### Flow Cytometry Analysis

Mice were systemically perfused, and spleens harvested and mashed through a 70 μm filter to create a single cell suspension. After a 15 minute incubation in red blood cell lysis buffer, cells were blocked for 30 minutes on ice with Fc block (anti CD16/CD32, BD Pharmingen) diluted 1:200 in 3% FBS in PBS. Cells were then stained with anti-F4/80-FITC, anti-Ly6C-PE, anti-CD11c-PerCP, and anti-CD11b-APC for 30 minutes, followed by 3 washes before being run on the LSRII.

### Luminex

Serum cytokines were measured using a Luminex Mouse Magnetic Bead Panel according to the manufacturer’s instructions (Millipore). Serum was tested in triplicate using a Bio-Rad Bio-Plex reader and analyzed using xMAP software.

### NGAL ELISA

Serum and urine samples were collected at sacrifice and analyzed for NGAL levels using the mouse-Lipocalin2/NGAL DuoSet ELISA from R&D systems (Minneapolis, MN), according to the manufacturer’s instructions.

### Statistics

To assess significant differences between groups, an ANOVA was performed, followed by multiple comparisons via Tukey’s Test.

## Additional Information

**How to cite this article**: Chalmers, S. A. *et al*. Therapeutic Blockade of Immune Complex-Mediated Glomerulonephritis by Highly Selective Inhibition of Bruton's Tyrosine Kinase. *Sci. Rep.* **6**, 26164; doi: 10.1038/srep26164 (2016).

## Supplementary Material

Supplementary Information

## Figures and Tables

**Table 1:** Molecular and cellular testing of BI-BTK-1.

Assay	Potency
BTK Enzyme IC_50_ (nM)	0.9 [0.6, 1.2]
BTK k_intact_/K_i_ (M^−1^ s^−1^)	85,000 ± 39,000
B-cell CD69 IC_50_ (nM)	2.4 [1.5, 3.8]
hWB CD69 IC_50_ (nM)	3.0 [1.7, 5.3]
Immune complex-Monocyte IL-6 IC_50_ (nM)	2.3 [0.9, 5.6]
Immune complex-Monocyte TNF-α IC_50_ (nM)	0.8 [0.4, 1.7]
Immune complex-Monocyte IL-1β IC_50_ (nM)	0.6 [0.3, 1.1]

IC50 values represent geometric mean [+/− one SD] for the following assays: BTK enzymatic activity (n = 3), purified human B cell CD69 (n = 5), human whole blood CD69 (n = 6), and immune complex mediated ativation of purified human monocytes (n = 7). BTK kinetic data represents geomean ± SD (n = 7).
