# Supplementary material for: Therapeutic Blockade of Immune Complex-Mediated Glomerulonephritis by Highly Selective Inhibition of Bruton’s Tyrosine Kinase
Source: Sci Rep. 2016 May 19;6:26164. doi: 10.1038/srep26164 (PMC4872164; doi:10.1038/srep26164)
Supplement: Supplementary Information [file srep26164-s1.pdf]

Supplemental Tables, Figures, and Figure Legends

Therapeutic Blockade of Immune Complex-Mediated Glomerulonephritis by Highly Selective Inhibition of Bruton's Tyrosine Kinase

By: Samantha A. Chalmers, Jessica Doerner, Todd Bosanac, Sara Khalil, Dustin Smith, Christian Harcken, Janice Dimock, Evan Der, Leal Herlitz, Deborah Webb, Elise Seccareccia, Di Feng, Jay S. Fine, Meera Ramanujam, Elliott Klein, and Chaim Putterman

Supplemental Table 1

Kinases inhibited by BI-BTK-1 with a BTK selectivity factor of less than 100-fold. Profiling against a panel of 282 kinases (Invitrogen Select Screen Kinase panel), BI-BTK-1 treatment resulted in significant inhibition (>80% inhibition at 3 µM) of 8 kinases: BTK, BLK, BMX, ERBB4, TEC, TXK, RET, PTK6. Of these, only BMX, TEC, TXK, PTK6 were inhibited with a BTK selectivity factor of less than 100-fold.

*Kinases that contain a cysteine residue aligning with Cysteine 481 in BTK.

Kinase	IC_{50} (nM)
**BTK***	0.9
**BLK***	300
**BMX***	2.3
**EGFR***	516
**ErbB2***	6350
**ErbB4***	42
**ITK***	>10000
**JAK3***	<10% inhibition @ 3µM
**TEC***	43.9
**TXK***	2.3
**PTK6**	37.0

Supplemental Table 2: Analysis of the top 50 pathways (FDR<0.01) that were enriched in the canonical pathway maps using MetaCore (Thomson Reuters).

Enrichment by Pathway Maps			
	**MAPS**	**p-value**	**Network objects from active data**
1	**Cell cycle_The metaphase checkpoint**	7.19054E-16	MAD2b, Rod, Aurora-B, HEC, Survivin, CENP-E, Nek2A, BUB1, CENP-A, SPBC24, Aurora-A, PLK1, CDCA1, CDC20, Zwilch, MAD2a, AF15q14, BUBR1
2	**Cell cycle_Role of APC in cell cycle regulation**	6.63918E-13	MAD2b, Tome-1, Cyclin A, Aurora-B, Kid, Cyclin B, CKS1, Nek2A, BUB1, Aurora-A, PLK1, CDC20, MAD2a, CDK1 (p34), BUBR1
3	**Cell cycle_Spindle assembly and chromosome separation**	3.48179E-10	Aurora-B, HEC, Kid, Tubulin alpha, Cyclin B, Separase, Nek2A, TPX2, Aurora-A, CDC20, MAD2a, CDK1 (p34), Tubulin (in microtubules)
4	**Immune response_Alternative complement pathway**	3.20106E-09	C7, iC3b, C3dg, Factor B, C3a, C3, C5 convertase (C3b2Bb), Factor I, Factor Ba, Annexin II, C3b, Factor Bb, C3 convertase (C3bBb), C3c, Clusterin
5	**Cell cycle_Chromosome condensation in prometaphase**	4.22531E-09	CAP-H/H2, Cyclin A, Aurora-B, Cyclin B, TOP2, BRRN1, CAP-G, CAP-G/G2, Aurora-A, CDK1 (p34)
6	**Cell cycle_Role of Nek in cell cycle regulation**	4.36961E-08	Tubulin beta, NEK6, HEC, Tubulin alpha, Nek2A, Cyclin B1, TPX2, Aurora-A, MAD2a, CDK1 (p34), Tubulin (in microtubules)
7	**IL-6 signaling in colorectal cancer**	2.38476E-07	STAT3, TGF-beta 1, Cyclin B, Survivin, ICAM1, Cyclin B1, c-Myc, Cyclin E, SOCS3, CDK1 (p34), Clusterin
8	**Glomerular injury in Lupus Nephritis**	2.83694E-07	ATF-4, CCL5, GRO-2, TGF-beta, VCAM1, PDGF-B, CX3CL1, IP10, CSF1, ICAM1, C3a, NGAL, Annexin II, A20, p38 MAPK, IFI56, FN14(TNFRSF12A)
9	**Hypertrophy of asthmatic airway smooth muscle cells**	4.5389E-07	STAT3, Smooth muscle myosin, Transgelin, ACTA2, TGF-beta 1, IFN-gamma receptor, EDNRB, PDGF-B, Endothelin-1, TGF-beta 3, c-Myc, MLCK, EDNRA, Actin muscle
10	**Fibroblast/ myofibroblast proliferation in asthmatic airways**	7.54636E-07	BDKRB1, TGF-beta 1, PAR1, EDNRB, CTGF, PKC, Tissue factor, Endothelin-1, ITGB3, des-Arg10-kallidin, EDNRA
11	**Immune response_Lectin induced complement pathway**	8.80272E-07	C2, C7, iC3b, C3dg, C2b, C3a, C3, Factor I, C3b, C2a, C3c, Clusterin
12	**Immune response_Classical complement pathway**	1.72125E-06	C2, C7, iC3b, C3dg, C2b, C3a, C3, Factor I, C3b, C2a, C3c, Clusterin
13	**Glomerular injury in Lupus Nephritis_draft**	2.61289E-06	ATF-4, CCL5, VCAM1, CX3CL1, IP10, CSF1, ICAM1, C3a, C3, NGAL, Annexin II, A20, p38 MAPK, IFI56, FN14(TNFRSF12A)
14	**Stem cells_Role of TGF-beta 1 in fibrosis development after myocardial infarction**	2.93989E-06	Biglycan, ACTA2, TGF-beta 1, EDNRB, CTGF, Endothelin-1, TIMP2, Tenascin-C, PAI1, EDNRA
15	**Blood coagulation_Blood coagulation**	3.80447E-06	Thrombomodulin, Fibrinogen gamma, Fibrinogen alpha, Tissue factor, Fibrinogen (fibrin), A2M, PAI1, Fibrinogen beta, Bradykinin, KNG
16	**Reproduction_Progesterone-mediated oocyte maturation**	4.8813E-06	CPEB1, CDC25C, BUB1, Cyclin B1, Aurora-A, PLK1, G-protein alpha-i family, CDC20, Kinase MYT1, CDK1 (p34)
17	**Cell cycle_Initiation of mitosis**	5.9149E-06	Cyclin B2, CDC25C, Cyclin B1, c-Myc, PLK1, MYRL2, Kinase MYT1, CDK1 (p34)
18	**Fenofibrate in treatment of type 2 diabetes and metabolic syndrome X**	5.9149E-06	APOA1, ABCA1, Fibrinogen gamma, Fibrinogen alpha, Fibrinogen (fibrin), PAI1, Fibrinogen beta, Acyl-CoA synthetase
19	**Stromal-epithelial interaction in Prostate Cancer**	7.84652E-06	ACTA2, TGF-beta 1, PDGF-B, Hic-5/ARA55, TIMP2, TGF-beta 3, Tenascin-C, Vimentin, Keratin 18, FGF10
20	**G protein-coupled receptors signaling in lung cancer**	1.27821E-05	CCL5, STAT3, HB-EGF, I-kB, Amphiregulin, BDKRB2, Galpha(q)-specific peptide GPCRs, EDNRB, LPA1 receptor, Endothelin-1, G-protein alpha-i family, EDNRA, Bradykinin
21	**Development_TGF-beta-dependent induction of EMT via RhoA, PI3K and ILK.**	1.86428E-05	I-kB, ACTA2, TGF-beta 1, Tropomyosin-1, Hic-5/ARA55, TGF-beta 3, Vimentin, Claudin-1, Actin, Cofilin
22	**Abnormalities in cell cycle in SCLC**	2.01842E-05	Cyclin A, Aurora-B, CKS1, p21, Cyclin B1, c-Myc, Cyclin E, CDK1 (p34)

Supplemental Table 2, continued

Enrichment by Pathway Maps (continued)			
	**MAPS**	**p-value**	**Network objects from active data**
23	**Cytoskeleton remodeling_Cytoskeleton remodeling**	2.60072E-05	PLAUR (uPAR), TGF-beta 1, Alpha-actinin, Filamin A, Alpha-actinin 1, Collagen IV, p21, c-Myc, p38 MAPK, PAI1, Zyxin, MLCK, MRLC, eIF4A, Cofilin
24	**Apoptosis and survival_Role of IAP-proteins in apoptosis**	3.44045E-05	c-IAP2, Survivin, Cyclin B1, c-IAP1, NAIP, tBid, CDK1 (p34), Bid
25	**Cytoskeleton remodeling_Neurofilaments**	6.07683E-05	Tubulin beta, Desmin, Nestin, Tubulin alpha, Vimentin, Peripherin, Tubulin (in microtubules)
26	**Cytoskeleton remodeling_TGF, WNT and cytoskeletal remodeling**	7.12495E-05	PLAUR (uPAR), TGF-beta 1, Alpha-actinin, Alpha-actinin 1, Collagen IV, p21, FRAT1, c-Myc, WNT, Actin, p38 MAPK, PAI1, MLCK, MRLC, Cofilin
27	**MAPK-independent proliferation of normal and asthmatic smooth muscle cells**	7.30357E-05	STAT3, HB-EGF, I-kB, TGF-beta 1, EDNRB, PDGF-B, p21, Endothelin-1, c-Myc, G-protein alpha-i family, EDNRA
28	**Development_Regulation of epithelial-to-mesenchymal transition (EMT)**	7.30357E-05	ACTA2, TGF-beta 1, Tropomyosin-1, PDGF-B, Endothelin-1, TGF-beta 3, Vimentin, Claudin-1, WNT, PAI1, EDNRA
29	**DNA damage_ATM / ATR regulation of G2 / M checkpoint**	8.01256E-05	Cyclin A, Brca1, Cyclin B, p21, CDC25C, Kinase MYT1, CDK1 (p34)
30	**Plasminogen activators signaling in pancreatic cancer**	8.83032E-05	HB-EGF, Galectin-1, PLAUR (uPAR), TGF-beta 1, Nek2A, Fra-1, Annexin II, PAI1
31	**Cytoskeleton remodeling_Keratin filaments**	0.000109346	Tubulin beta, Keratin 8, Tubulin alpha, Vimentin, Keratin 18, Keratin 8/18, CDK1 (p34), Tubulin (in microtubules)
32	**MAPK-mediated proliferation of normal and asthmatic smooth muscle cells**	0.000111028	HB-EGF, TGF-beta 1, Amphiregulin, EDNRB, PDGF-B, LPA1 receptor, Endothelin-1, PAI1, G-protein alpha-i family, EDNRA
33	**Eosinophil adhesion and transendothelial migration in asthma**	0.000129092	CCL5, Galectin-3, PLAUR (uPAR), Osteopontin, VCAM1, PKC, Collagen IV, ICAM1, Fibrinogen (fibrin), C3a, p38 MAPK
34	**Role of Th17 cells in asthma**	0.000133913	HB-EGF, GRO-2, TGF-beta 1, VCAM1, ICAM1, p38 MAPK, MRLC
35	**Development_TGF-beta-dependent induction of EMT via MAPK**	0.000139644	TGF-beta 1, TGF-beta, Endothelin-1, TGF-beta 3, Vimentin, ITGB3, Claudin-1, p38 MAPK, PAI1
36	**TGF-beta signaling via kinase cascades in breast cancer**	0.000150729	TGF-beta 1, Amphiregulin, p38beta (MAPK11), Survivin, TIMP2, Neuregulin 1, ITGB3, p38 MAPK, PAI1, NFKBIA
37	**TNF-alpha-induced inflammatory signaling in normal and asthmatic airway epithelium**	0.000163928	CCL5, I-kB, CCL17, IP10, ICAM1, p38 MAPK, NFKBIA, FN14(TNFRSF12A)
38	**Stimulation of TGF-beta signaling in lung cancer**	0.000165476	I-kB, ACTA2, TGF-beta 1, TGF-beta, Tropomyosin-1, TGF-beta 3, Vimentin, PAI1, Tropomyosin-2
39	**IFN-gamma and Th2 cytokines-induced inflammatory signaling in normal and asthmatic airway epithelium**	0.000198638	CCL5, IL-2R gamma chain, IFN-gamma receptor, CCL17, IP10, ICAM1, p38 MAPK, SOCS3
40	**Role of stellate cells in progression of pancreatic cancer**	0.000201772	HB-EGF, GRO-2, Galectin-1, NGF, ACTA2, TGF-beta 1, PDGF-B, CTGF, Fibrinogen (fibrin), NFKBIA
41	**Cell adhesion_Gap junctions**	0.000213802	Tubulin beta, Tubulin alpha, PKC, Actin, Connexin 31, Connexin 43, Tubulin (in microtubules)
42	**Development_Transcription factors in segregation of hepatocytic lineage**	0.000213802	Activin B, TGF-beta 1, GGTL3, p21, A2M, FGF10, Activin
43	**Expression targets of Tissue factor signaling in cancer**	0.000243842	PLAUR (uPAR), PAR1, CTGF, CSF1, Tissue factor, PAI1
44	**SHH signaling in colorectal cancer**	0.000266126	GLI-3R, GLI-3, p21, PTCH1, Vimentin, c-Myc, WNT
45	**Cell adhesion_ECM remodeling**	0.000311983	HB-EGF, PLAUR (uPAR), Stromelysin-2, Collagen IV, TIMP2, MSN (moesin), Nidogen, Osteonectin, PAI1
46	**Cell adhesion_Chemokines and adhesion**	0.000316106	PLAUR (uPAR), Alpha-actinin, Filamin A, Alpha-actinin 1, Collagen IV, MSN (moesin), c-Myc, Actin, PAI1, G-protein alpha-i family, Zyxin, CD47, Cofilin
47	**Protein folding and maturation_Bradykinin / Kallidin maturation**	0.000328271	BDKRB1, Kallidin, des-Arg9-bradykinin, BDKRB2, des-Arg10-kallidin, Bradykinin, KNG
48	**TGF-beta-induced fibroblast/ myofibroblast migration and extracellular matrix production in asthmatic airways**	0.000348006	Biglycan, TGF-beta 1, COL4A1, Collagen IV, Endothelin-1, TIMP2, TGF-beta 3, Tenascin-C, p38 MAPK, PAI1
49	**Notch signaling in breast cancer**	0.000361849	NOXA, Cyclin A, HURP, Survivin, p21, Cyclin B1, HEYL, c-Myc, Mcl-1
50	**Renal tubulointerstitial injury in Lupus Nephritis**	0.000395899	CCL5, I-kB, TGF-beta 1, VCAM1, IFN-gamma receptor, CSF1, ICAM1, Vimentin, PAI1, FN14(TNFRSF12A)

Supplemental Figure 1

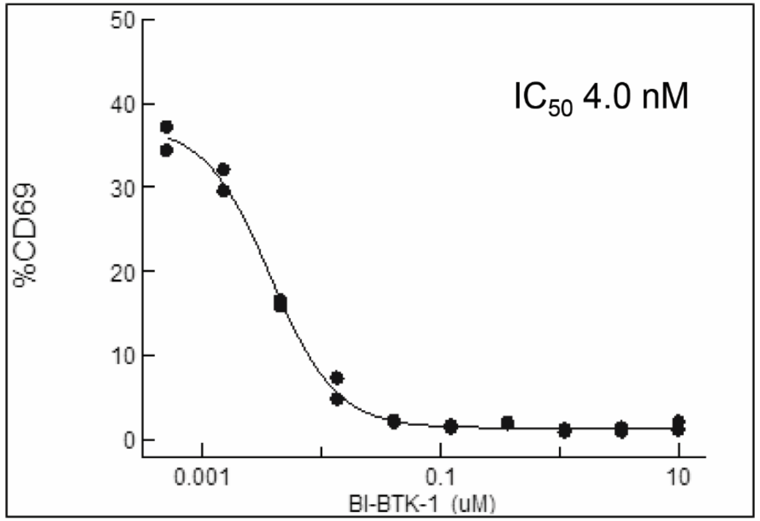

Supplemental Figure 2

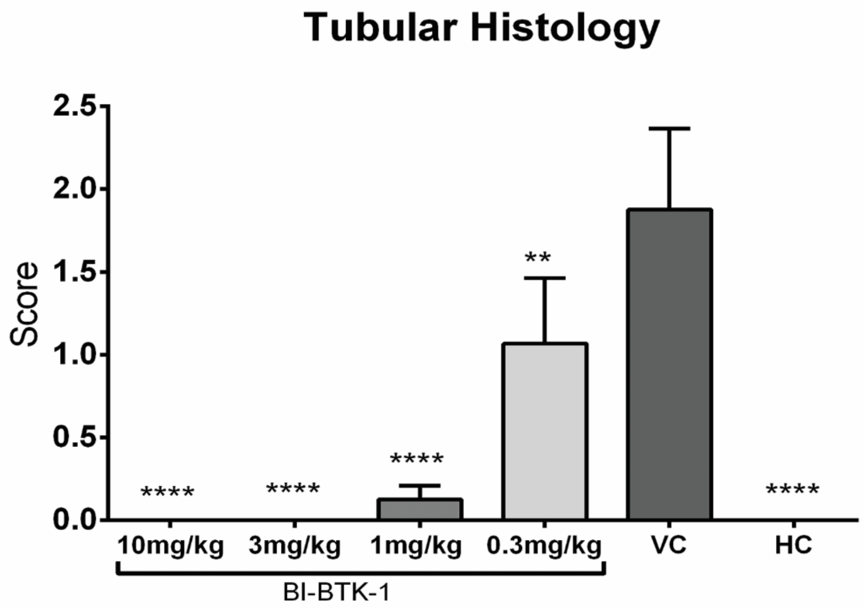

Supplemental Figure 3

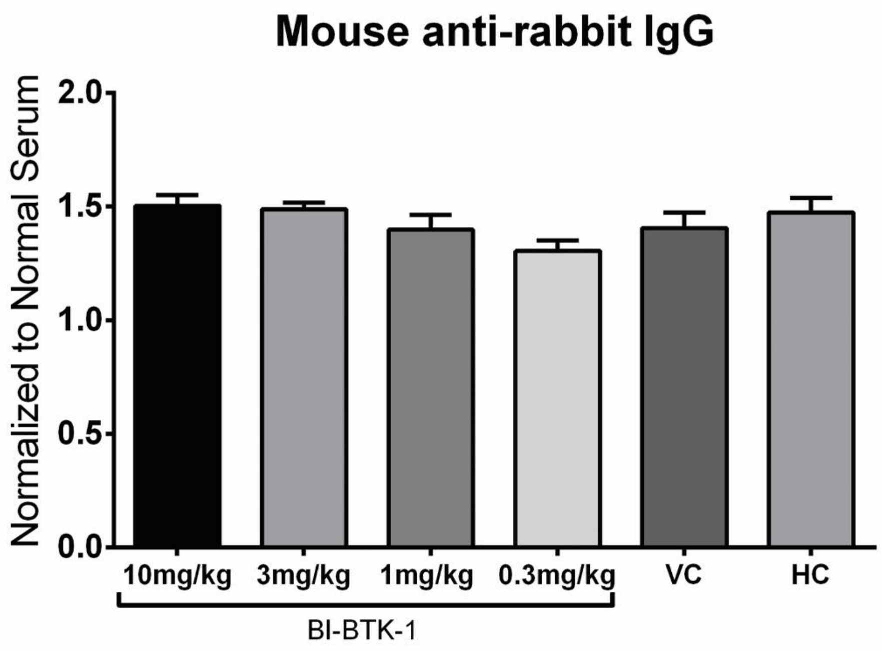

Supplemental Figure 4

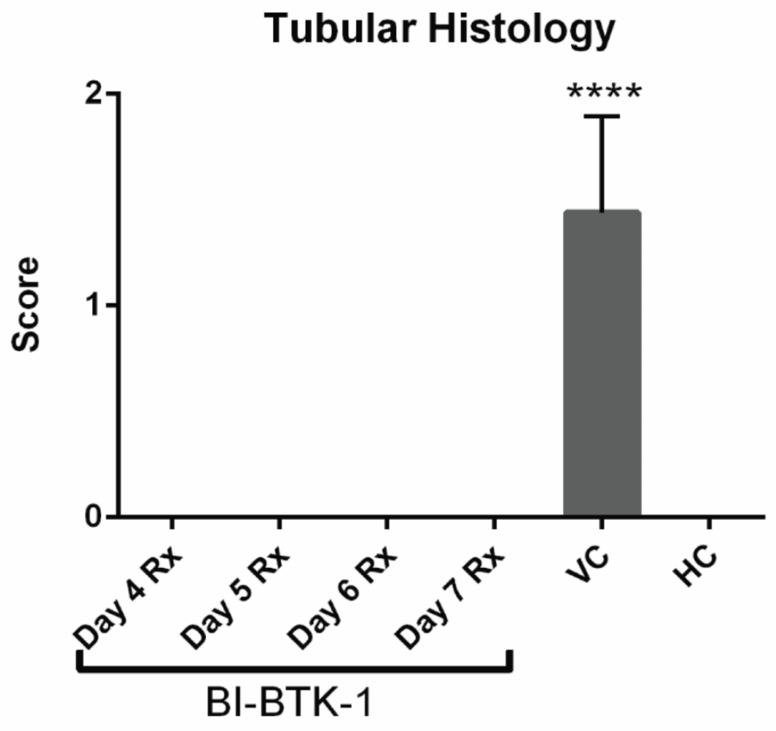

SUPPLEMENTAL FIGURE LEGENDS

Supplemental Figure 1. Cellular activity of BI-BTK-1. Inhibition of αIgD stimulated CD69 expression on primary human CD19+ B cells from human whole blood. Percentage of CD69+ cells as determined by flow cytometry is presented.

Supplemental Figure 2. Renal histopathology. Tubular histology scores are shown from the dose responsive BI-BTK-1 NTN experiment. Shown here are results from one experiment (10 mg/kg BI-BTK-1 treated, n=8; 3 mg/kg BI-BTK-1, n=8; 1 mg/kg BI-BTK-1, n=8; 0.3 mg/kg BI-BTK-1, n=8; VC, n=8; HC, n=5). Asterisks represent a significant difference compared to VC (**p<0.01, ****p<0.0001).

Supplemental Figure 3. Disease induction checkpoints. BI-BTK-1 did not interfere with the day 0 immunization, as assessed by mouse anti-rabbit IgG levels (B) in the terminal serum. Shown here are results from one experiment (10 mg/kg BI-BTK-1 treated, n=8; 3 mg/kg BI-BTK-1, n=8; 1 mg/kg BI-BTK-1, n=8; 0.3 mg/kg BI-BTK-1, n=8; VC, n=8; HC, n=5). Asterisks represent a significant difference of HC compared to all other groups (*p<0.05).

Supplemental Figure 4. Renal histology in the delayed treatment of NTN with BI-BTK-1. Shown are the tubular histology scores from one experiment. (day 4 start, n=8; day 5 start, n=8; day 6 start, n=8; day 7 start, n=8; VC, n=8; HC, n=5). Asterisks represent a significant difference of VC compared to all other groups (*p<0.05, **p<0.01, ***p<0.001, ****p<0.0001).
